# Complex study on compression of ECG signals using novel single-cycle fractal-based algorithm and SPIHT

**DOI:** 10.1038/s41598-020-72656-6

**Published:** 2020-09-25

**Authors:** Andrea Nemcova, Martin Vitek, Marie Novakova

**Affiliations:** 1 Department of Biomedical Engineering, Faculty of Electrical Engineering and Communication, Brno University of Technology, Technická 12, 616 00 Brno, Czech Republic; 2 Department of Physiology, Faculty of Medicine, Masaryk University, Kamenice 753/5, 625 00 Brno, Czech Republic

**Keywords:** Biomedical engineering, Engineering, Electrophysiology, Sensors and probes

## Abstract

Compression of ECG signal is essential especially in the area of signal transmission in telemedicine. There exist many compression algorithms which are described in various details, tested on various datasets and their performance is expressed by different ways. There is a lack of standardization in this area. This study points out these drawbacks and presents new compression algorithm which is properly described, tested and objectively compared with other authors. This study serves as an example how the standardization should look like. Single-cycle fractal-based (SCyF) compression algorithm is introduced and tested on 4 different databases—CSE database, MIT-BIH arrhythmia database, High-frequency signal and Brno University of Technology ECG quality database (BUT QDB). SCyF algorithm is always compared with well-known algorithm based on wavelet transform and set partitioning in hierarchical trees in terms of efficiency (2 methods) and quality/distortion of the signal after compression (12 methods). Detail analysis of the results is provided. The results of SCyF compression algorithm reach up to avL = 0.4460 bps and PRDN = 2.8236%.

## Introduction

The general development of information and communication technologies manifests in medicine as well and contributes to improvement of health care worldwide. It is very important nowadays when we are facing fast ageing of population^1^, increasing number of chronic diseases^2^ and thus higher press on healthcare providers. The most common cause of death worldwide are cardiovascular diseases (CVD)^3^. According to World Health Organization (WHO)^3^, CVD killed 17.9 million people in 2016 which represents 31% of worldwide deaths. Thus, the early detection and treatment of people with risk factors is important. For this purpose, telemedicine, especially its third largest area—patient remote monitoring^4^—is used. Within remote patient monitoring area, there belong electrocardiogram (ECG) recording. ECG is considered a noninvasive gold standard for identification of many health problems such as cardiac arrhythmias, bundle branch blocks, coronary artery disease, ventricular hypertrophy, heart failure, and many others^5^.

For efficient transmission of data between patient and doctor or between two doctors or hospitals and medical centers, compression is necessary. The aim of compression is to reach maximum efficiency of data reduction without loss of diagnostic information.

Transmission in telemedicine can be real-time (synchronous)—the patient is monitored by the doctor 24 h a day (small delay may be accepted—e.g. for compression); or store-and-forward (asynchronous)—the data are stored and transmitted when the internet connection is accessible^4,6^. Thanks to the compression, the transmission is faster^7^, it has lower energy consumption (especially in case of wireless transmission)^8,9^, thus the device has prolonged battery life^10^, and transmitted data needs less storage capacity^7,11^. Lower energy consumption leads to miniaturization of mobile devices. The channel bandwidth is limited; thus the compression is necessary for high volume data^6,12^. Compression can be utilized for archiving of data or even big data^13^.

To gain significant data reduction and minimize power consumption in ECG monitoring, lossy compression is preferred^8^ to lossless variant. Lossy compression is always connected with information loss. However, good compression algorithm should not lose or distort diagnostic information. As a matter of principle, compression is always compromise between size of the data and their quality^14^. Therefore, the assessment of ECG signal quality after compression and the determination of compression efficiency should be an essential part of compression itself^15^.

There exist many methods for ECG signal compression. These methods work with various successfulness and they are based on various principles. Many of them use intra-beat, inter-beat and/or inter-channel correlation of ECG signals^12^. Lossy methods are divided into one-dimensional (1D) and two-dimensional (2D) groups^12^. 1D methods split in three groups—direct compression methods, transform-domain methods and parameter extraction methods^16^. Some compression methods combine several basic principles and therefore cannot be included in one single group. Short survey on some recent compression methods follows.

Compression based on wavelet transform (WT) is popular and efficient^12^. There belong e.g. method based on WT and Set Partitioning in Hierarchical Trees (SPIHT) algorithm originally published by Lu^13^ and modified by Hrubes^17^. Agulhari^18^ combined WT with threshold based selection of significant coefficients, their quantization and modified run-length encoding (RLE). Bilgin^19^ modified WT-based JPEG2000 algorithm to enable ECG data compression. Pandey^20^ recently introduced compression algorithm based on JPEG2000 and 2D discrete cosine transform. Jha^21^ started the compression procedure with filtering, continued with backward signal difference, WT, thresholding and quantization, and ended with RLE. Padhy^22^ used WT in combination with Singular Value Decomposition (SVD) and energy-based thresholding for multilead ECG compression. Bera^23^ used hybrid compression of ECG starting with SVM-based binary classifier of ECG beats to normal and abnormal. He continued with wavelet-based compression of abnormal beats and combined wavelet- and Principal Component Analysis (PCA)-based compression of normal beats. Fractal-based algorithms (e.g. Khalaj^24^, Lin^11^, Ibaida^25^) are considered highly efficient^11^. Fractal-based methods are used also for image^26^ and video^27^ compression. Nowadays, methods based on Compressed Sensing (CS) are very popular and perspective. Balestrieri^28^ introduced a variant of CS method suitable for efficient real-time ECG acquisition in the Internet of Medical Things area. Adamo^29^ combined CS with dictionary based method using *k*-LiMapS algorithm to obtain sparse vector and Huffman coder to encode sparse coefficients. Singh^30^ combined PCA, CS, quantization and Huffman encoding for multilead ECG compression. Nasimi^31^ introduced selective compression method which is based on assessing the similarity between consecutive segments (beats). If the segments are similar, compression is provided using CS and if the segments are not similar it points to some pathology and thus the segment is preserved and transmitted without compression. Other authors combined Empirical Mode Decomposition with encoding of extracted parameters (Khaldi^32^), extraction of extrema points (Zhao^33^), or WT (Wang^34^, Jha^35^). Following methods are based on various principles. Elgendi^9^ used for compression simple scheme—combination of low-pass filter and downsampling by factor K. For ECG compression, Ma^36^ introduced combination of Adaptive Fourier Decomposition (AFD) and symbol substitution. Fira^37^ developed compression method where local minima and maxima are extracted. These points are called skeleton. Skeleton is further optimized by discarding and adding appropriate samples using thresholding. To enhance the efficiency of this compression algorithm, delta and Lempel–Ziv–Welch (LZW) coding is applied. Lee^14^ introduced compression method based on peak-to-peak segmentation, Discrete Cosine Transform (DCT), window filtering, and Huffman coding. Tan^38^ recently introduced Blaschke unwinding adaptive Fourier decomposition method. Boom of deep learning manifests in compression as well—Wang^39^ introduced promising deep learning based Spindle Convolutional Auto-Encoder (SCAE) method.

More complex reviews on compression methods can be found in studies by Jalaleddine^40^ (1990), Manikandan^12^ (2014), Kale^16^ (2016), Rajankar^41^ (2018), Tiwari^42^ (2019). Summary of quality assessment of ECG signals after compression are in reviews by Dandapat^43^ (2015) and Nemcova^15^ (2018).

There is a lack of standards for evaluation of ECG signal quality after compression^44^. Compression algorithms should be tested on the whole standard ECG databases including all signals and all leads^22,30^. However, the usual practice is that authors test their algorithms on selected and/or shortened signals/leads^11,13,17–19,24,29,33,34,36,37^ from standard or even non-standard databases. In some studies, the tested database^25,32^ or detail information such as number of tested signals^25^ or number and type of leads^9,14,21,34^ of the signals on which the algorithms were tested are missing. Some studies tested their algorithm on one signal only^32^. In the growing Internet of Things (IoT) era, there is growing need to focus on ECGs sensed by up-to-date devices under free-living conditions. Such data were sensed by us and used in this study; the data are described in Data section as a Brno University of Technology ECG quality database (BUT QDB). In many studies, the authors use Percentage Root Mean Square Difference (PRD) to evaluate quality of the ECG signal after compression and reconstruction. However, many of them do not subtract the offset and/or DC component and thus the results are artificially better and objective comparison with other authors is irrelevant. This topic was discussed in our previous study^15^ where the details and recommendations for quality assessment of ECG signal after compression and reconstruction can be found.

The first aim of this study is to point out drawbacks, gaps, and mistakes present in the area of compression to make compression results objective, reliable and to enable objective comparison between authors. The second aim of the study is to introduce new Single-Cycle Fractal-Based (SCyF) compression method and to compare it with WT + SPIHT based method. Description of the SCyF algorithm as well as the testing scheme and results prevent general compression drawbacks described in this study and serves as an example how the standardization should look like.

## Methods

### Data

In this study, four various databases of ECG signals are used. They differ in recording device, number of signals and leads, sampling frequency, duration, bit resolution and signal content (noise, pathologies, artifacts).

A. Common Standards for Quantitative Electrocardiography (CSE) database^45,46^ is a standard database which contains 125 signals, each including 15 leads (12 standard and 3 Frank leads). The duration of each signal is 10 s, sampling frequency is 500 Hz, and bit resolution is 16 bits per sample (bps). Signals no. 67 and 70 contain pacemaker peaks. Signals no. 86–97 have a duration of 8 s, thus the rest 2 padded seconds were cut. Three Frank leads of signals no. 60, 68, 76, 84, 92, 100, 108, and 124 which are not ECG signals (include constant signal) were excluded. It is not effective to compress constant signals by compression algorithm which is designed for ECG signals. It will be much more efficient and faster to use RLE of constant segments before the compression of ECG itself.
B. Massachusetts Institute of Technology–Beth Israel Hospital (MIT-BIH) arrhythmia database (MITADB)^47,48^ is the most cited standard database^46^. It consists of 48 half-hour ECG signals, each including 2 leads. The sampling frequency is 360 Hz, bit resolution 11 bps.
C. High-frequency signal was recorded by the International Clinical Research Center of St. Anne’s University Hospital Brno in cooperation with the Institute of Scientific Instruments of the Czech Academy of Sciences^49,50^. In this study, one signal containing 19 leads is used. The sampling frequency is 5000 Hz, bit resolution is 24 bps, and duration is ca. 21 min. The signal is further called UPT signal. This signal is not publicly available.
D. Our own experimental database of ECG signals (BUT QDB)^47,51^ was recorded using Bittium Faros 180. In this study, three single-lead signals from this database are used. The duration of each signal is minimally 24 h, the sampling frequency is 1000 Hz, bit resolution is 16 bps. The signals were sensed from 3 different people under free-living conditions. The signals were manually annotated by 3 ECG experts in terms of the quality of the signal. The first class contains segments suitable for full-wave analysis; the second class includes segments suitable for reliable QRS detection and heart rate variability (HRV) analysis; the third-class segments are not recommended for any reliable analysis. The data are publicly available on Physionet.

### Compression algorithm based on WT and SPIHT

As a well-known, advanced, efficient, and popular compression algorithm, WT based method in combination with SPIHT is used. This method is considered as a gold standard for the purpose of this study.

SPIHT is the progressive iterative compression algorithm. The output bit flow can be controlled by the user in terms of Average Value Length (avL) or Normalized Percentage Root Mean Square Difference (PRDN). SPIHT uses the Temporal Orientation Tree, where one wavelet coefficient in lower frequency bands corresponds to two wavelet coefficients (offspring) in higher frequency bands or has no offspring. Individual coefficients or the whole trees are coded according to their significance (threshold is used). The detail principle is difficult to explain; thus, it is only briefly sketched in here, details and example can be found in^13^. At first, WT (discrete time WT using wavelet filter bank bior4.4 and decomposition level 6)^17^ is performed in frame of ECG (length 1024 samples)^17^ and Temporal Orientation Tree is created. Then the first threshold is calculated and List of Insignificant Sets (LIS), List of Insignificant Points (LIP), and List of Significat Points (LSP) are initialized. After that sorting pass in LIP, sorting pass in LIS, and refinement pass are performed. Then the threshold is reduced, and the process repeats until the criterium (desired effectivity or quality) is met.

In this study, algorithm described and implemented by Hrubes^17^ and on 1D signals originally introduced by Lu^13^ is used. Moreover, this algorithm is based on different principle than newly introduced SCyF method, which supports objectivity of this study.

### SCyF compression method

As the basis for the SCyF compression algorithm, the fractal-based one by Ibaida^25^ was chosen. The reason is great compression efficiency (Compression Factor (CF) = 42) and low compression error (PRD < 1%). During implementation of this method in Matlab, a few mistakes were found. The equations for calculation of scale coefficient and Fractal Root Mean Square (FRMS) are inaccurate in the original article^25^. Correct equations are in the article by Al-Shammary^52^. Ibaida^25^ used not-normalized equation for calculation of the PRD and did not mention the DC component and the offset subtraction. It means that the PRD error is artificially lower than the normalized PRD (PRDN) as proved by Nemcova^15^. Ibaida^25^ also did not mention which signal(s) from MITADB they used to reach the results mentioned above.

**Figure 1:**
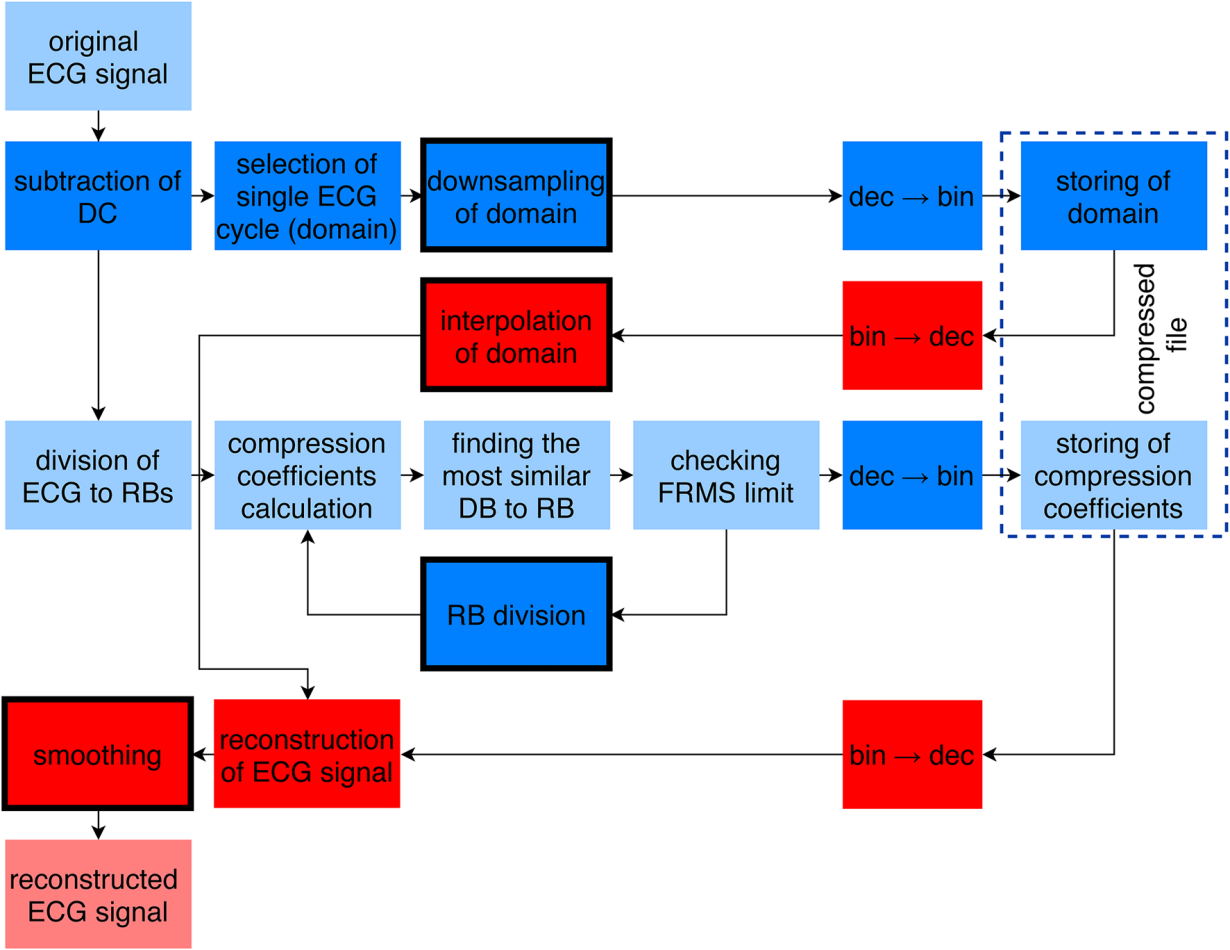
Block diagram of SCyF compression method. Blue color is for compression and red one for reconstruction part of the method. Blocks that are new against Ibaida’s^25^ work, are saturated and optional ones have thick solid border. Dashed line borders blocks that create compressed file.

SCyF compression method is based on Ibaida’s^25^ method with correct equations by Al-Shammary^52^. SCyF algorithm utilizes self-similarity of ECG signal. The principle is shown in Fig. 1. At first, as a preprocessing step, the DC component (including offset) is subtracted from the original signal.

### Domain

The algorithm utilizes the quasiperiodicity of the ECG signal. Single cycle of the currently compressed ECG is picked as a domain. Single cycle is selected manually as the most representative cycle of the beginning of currently compressed ECG signal. It starts with the beginning of P wave and ends just before the beginning of the following P wave. Domain is divided into overlapping blocks called domain blocks (DB) of size called block size (BS). Overlapping is controlled by predefined jump step (JS), which is the distance between two adjacent DB. BS should be smaller (usually) or equal to the length of the domain.

### Compression

**Figure 2:**
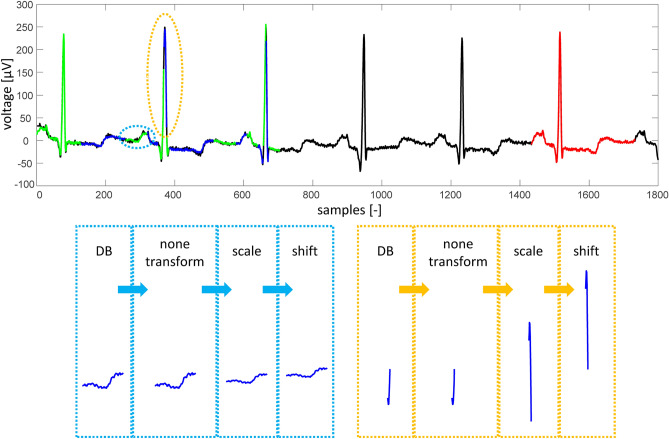
Transforming domain block to be as much similar as possible to the range block. Upper picture shows original ECG signal (black), domain (red), and compressed and reconstructed range blocks (dark blue and green; two adjacent RBs have different color). Two lower images show transformation process of domain block to be as much similar to the range block as possible. Transformation consists of affine/none transform (in this case none transform), application of scale coefficient (changes the size and ratio of the block) and application of shift coefficient (changes the offset of the block).

ECG signal is divided into non-overlapping blocks called range blocks (RB) which are of equal length BS. Then for each RB the most similar DB is searched. As a similarity metric, FRMS^52^ is used. To enhance the similarity between RB and DB, DB shape can be optimized using fractal coefficients and affine transform. In this study, two fractal coefficients are used: shift (offset) and scale^25,52^; and either affine transform (swapping each two adjacent samples) can be used or the block is not transformed (none transform). In our study, only one affine transform and none transform are used instead of four used by Ibaida^25^. The reasons are: a) the selected affine transform or none transform are the most common according to the testing we provided; b) it enhances the efficiency of compression because one type of affine transform and information about no transform can be expressed by one bit; c) it shortens the time of compression. If the most similar transformed DB still does not meet predefined FRMS limit, RBs are divided into two halves (each of length of ½ BS) and each one is compressed separately. This idea was published by Khalaj^24^. It means that for each smaller RB the most similar DB of the same length (½ BS) is searched. Transformations are done in the same manner as on larger block. If the criterion is still not met, RB can be further divided. Maximum number of divisions is preset by the user and it also depends on the initial setting of BS. Ideally, BS is set to 2^n^, where *n* is integer and BS is smaller or equal to the length of the domain. The higher the number of divisions, the higher quality of the signal after compression and reconstruction but also higher computational demand necessary for compression and lower effectivity. If the FRMS is not below the limit after the last division, the algorithm saves the best transform settings (with the lowest FRMS) for the shortest ranges (for the last possible division). Divided and already transformed RBs are drawn in Fig. 2 in green and dark blue color (two colors are used for better differentiation between adjacent blocks). The process of adjusting RB to be as much similar as possible to DB using fractal coefficients and affine transform is shown in Fig. 2 in blue and yellow borders. The domain is highlighted in red.

### Reconstruction

The reconstruction is provided in reverse manner using all outputs in single iteration unlike the original method^25^ which uses 4 iterations. Thus, the reconstruction is faster and less computational demand. On the DB with stored index, the transform coefficients are applied and the reconstruction RB is created. RBs are then joined into one signal.

Inputs of the SCyF algorithm are: ECG signal, BS, JS, FRMS limit, maximum number of block divisions and the beginning and the end of the single cycle of ECG.

Outputs of the compression part of the algorithm are: domain, index of DB, shift coefficient, scale coefficient, type of transform (affine/none) and number of divisions (all in binary form).

### Optional modifications

The domain can be computed as a downsampled version of the original signal. It means that two adjacent samples are averaged. Thus, the domain occupies less storage space, and the signal is smoother. Smoother signal means less noise but usually also distortion of high-frequency details (such as lowering of R peak).

The signal can be smoothed after compression and reconstruction to improve the connections between reconstructed RBs. For smoothing, the best method seems to be local regression using weighted linear least squares and a 2nd degree polynomial model. In some signals, smoothing improves the quality of the signal after compression and reconstruction and in some signals the quality is worsen.

Especially shorter signals can be extended on both sides (e.g. 10 samples on each side) to reach higher fidelity. It is balanced by higher computational demand, duration of compression and the amount of stored data.

SCyF algorithm is further compared with the original algorithm by Ibaida^25^. Because the original version is not publicly available, we implemented the algorithm on our own. The algorithm was implemented as much similarly as described in the article. However, we avoided mistakes in FRMS and scale coefficient. BS and JS were set according to the authors to 35 and 10, respectively. Ibaida^25^ recommended 4 iterations for reconstruction process. Our results are shown for 4 iterations (which seems to be not enough) and 10 iterations (which is more than the authors recommended but the signal has better quality).

### Evaluation of compression efficiency and quality of the reconstructed signal

Compression efficiency was calculated as CF and avL. CF is in some studies called Compression Ratio (CR), but we follow recommendations and definitions from our previous study^15^. Average Value Length^15^ (avL) is calculated as a ratio between bit size of the output stream (compressed ECG) and length of the original signal in samples. The unit is bits per sample (bps). avL informs about the number of bits that are needed on average to compress one sample of the signal. For assessment of ECG signal quality after compression, altogether 12 methods we previously recommended^15,53^ were used. These methods are namely: percentage similarity using standard deviation of normal-to-normal intervals (PSim SDNN), quality score (QS), signal to noise ratio (SNR), mean square error (MSE), PRDN1, maximum amplitude error (MAX), standard error (STDERR), wavelet-energy based diagnostic distortion using stationary wavelet transform (WEDD SWT), spectra difference (Spectrum), similarity—positions with tolerance of 10 (SiP10), similarity—positions and amplitude with tolerance of 10 (SiPA10), and dynamic time warping—percentage match of fiducial points (DTW pmfp2). Details about these methods can be found in^15,53^.

### The testing scheme

According to the aims of the study, the step by step testing scheme was created. The order was set according to the database which was used for testing. In the first two points, all four databases were used. In points 3–5, 6–7, 8–10, 11–12, the CSE database, MITADB, UPT signal and BUT QDB were used, respectively. The “Results and Discussion” chapter is divided accordingly.

1. Comparison of two compression algorithms based on different principles: WT + SPIHT and SCyF compression algorithms (all 4 databases).
2. Testing two variants of SCyF algorithm—with and without smoothing (all 4 databases).
3. Comparison of compression of 15 leads (CSE database).
4. Testing the compression algorithms on short (10 s) signals (CSE database).
5. Comparison of the results on the whole database and the database without two signals with pacemaker peaks (CSE database).
6. Comparison of the results with other authors (MITADB).
7. Comparison of the original fractal-based algorithm (with minimum changes) with the SCyF one (MITADB).
8. Testing the algorithms on high-frequency high-resolution signals (UPT signal).
9. Comparison of compression of 19 leads of high-frequency signal (UPT signal).
10. Testing how sampling frequency influences effectivity and quality of compression (UPT signal).
11. Testing the algorithms on free-living data (BUT QDB).
12. Comparison of effectivity and quality of compression in terms of the quality of the original signal (BUT QDB).

At first, the signals are compressed using SCyF algorithm. The settings of the SCyF algorithm are shown in Table 1. “Divisions” in the Table 1 stands for maximum number of block divisions. The SPIHT algorithm was controlled in terms of avL which was set individually for each signal according to the output avL of SCyF method.

**Table 1:** Settings of the SCyF algorithm for each database.

Database	BS	JS	FRMS limit	Divisions
CSE	256	1	12	2
MITADB	128	1	5	3
UPT signal	256	1	12	2
BUT QDB	256	1	12	2

To test how the sampling frequency influences effectivity and quality of compression, it was necessary to downsample the high-frequency UPT signal. Downsampling was performed 5 times to create signals with sampling frequencies of 2500 Hz, 1000 Hz, 500 Hz, 250 Hz, and 125 Hz.

## Results and discussion

### Comparison of two compression algorithms based on different principles (all 4 databases)

The numerical results in terms of compression efficiency and quality of the signal after compression and reconstruction are shown in Table 2. The rows of the Table 2 show compression algorithms (SPIHT means WT + SPIHT, SCyF and SCyF S are single-cycle fractal-based methods without and with smoothing, respectively, P means dataset with signals no. 67 and 70 in case of the CSE database). The rows are grouped according to the database on which the compression algorithms were tested. The columns show sampling frequency (fs), original bit resolution of the signal (bit res), avL and CF as measures of effectivity of the compression and 12 methods named in “Methods” section as measures of the quality or distortion. The green and yellow colors highlight whether the results are better for WT + SPIHT or SCyF method, respectively. It is evident that the SPIHT algorithm in most cases beats the SCyF algorithm for majority of quality indexes. However, there are some cases when the SCyF algorithm is better; these are: WEDD SWT metric, sometimes PSim SDNN and DTW pmfp2 and highly downsampled signal (fs = 125 Hz). The avL and CF are slightly better for SCyF algorithm because the SPIHT algorithm was set according to it.

**Table 2:**
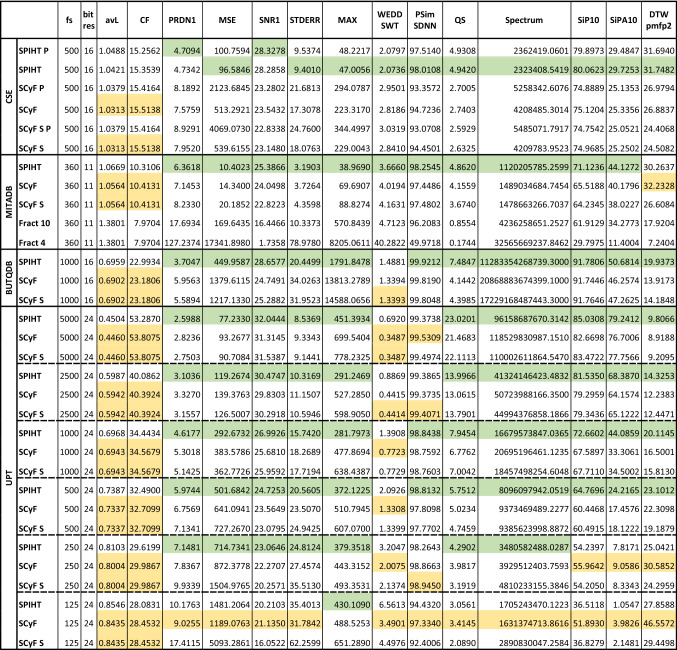
Results of testing WT + SPIHT and ScyF compression algorithms on four different databases.

**P** stands for the whole CSE database including pacemaker signals no. 67 and 70; S stands for smoothing. For MITADB, there are also results for original fractal-based algorithm implemented according to Ibaida^23^. These results are named Fract 10 and Fract 4, where the number stands for iterations needed for reconstruction.

### Testing of two variants of SCyF algorithm—with and without smoothing (all 4 databases)

The difference between SCyF algorithm without and with smoothing is not easily describable (as can be seen in Table 2). Sometimes smoothing improves the quality of the signal and in some cases, algorithm without smoothing reaches better results. Therefore, this block remains as an optional modification of the algorithm and it is not a core part of it.

### Comparison of compression of 15 leads (CSE database)

**Figure 3:**
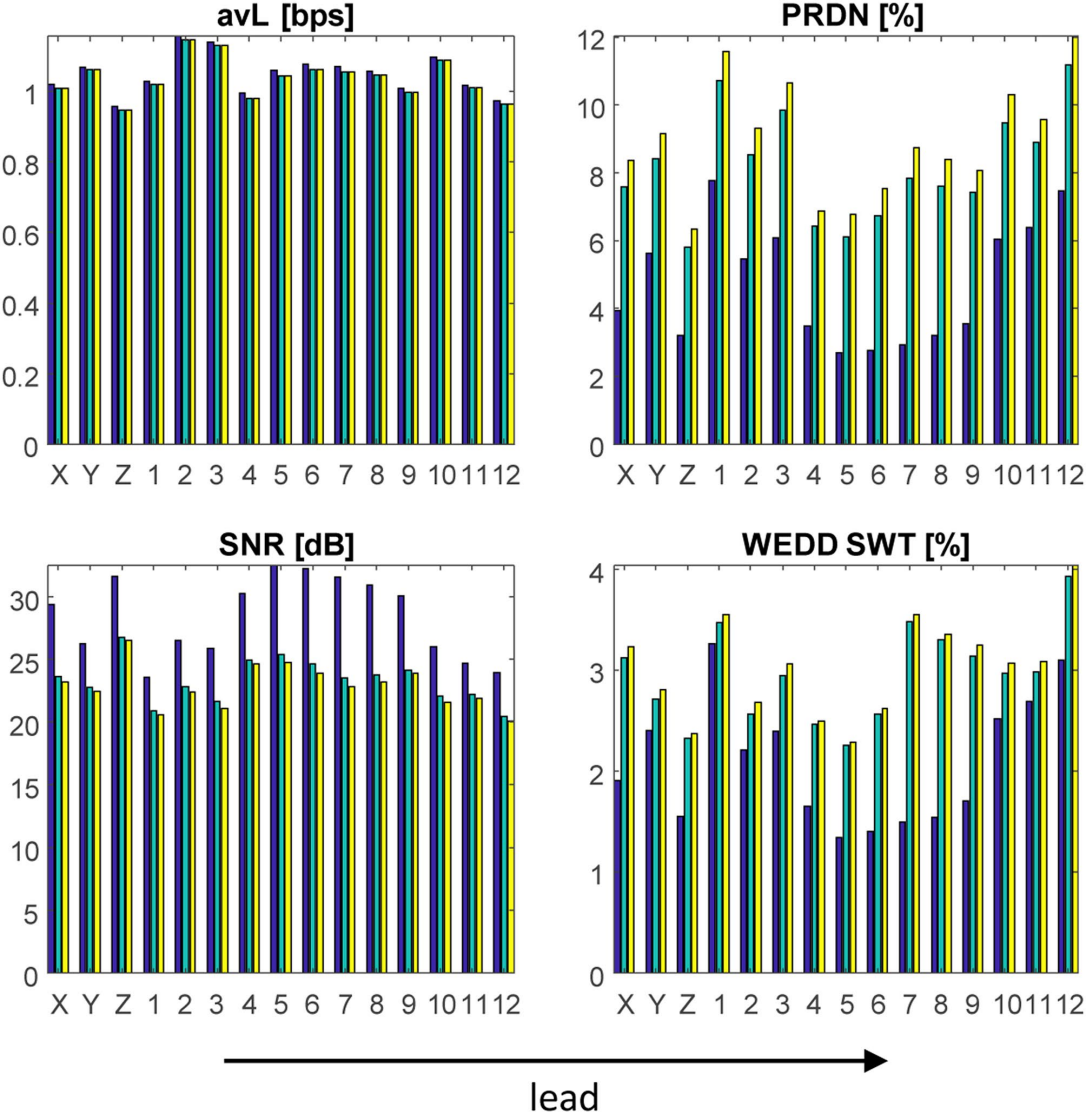
Results of testing WT + SPIHT and SCyF algorithms on the 15 leads of signals from the CSE database (x axis) including signals 67 and 70. Blue color represents the results of WT + SPIHT algorithm, green and yellow colors represent results of SCyF method without and with smoothing, respectively. For clarity, only 4 representative graphs were selected; complete results are in Supplementary Fig. S1 online.

Figure 3 shows the results of testing WT + SPIHT and SCyF algorithms on 15 leads of signals from the CSE database including signals no. 67 and 70 with pacemaker peaks. From Fig. 3, it is evident that various leads of CSE database are compressed with different effectivity and quality. In Fig. 3, there are only 3 selected methods of quality assessment and one method for efficiency assessment (avL); results of all 12 quality assessment methods are included in Supplementary Fig. S1 online. Both algorithms have similar trend of the results of effectivity and quality, although the principles of both algorithms are different. It means that compression performance is probably dependent on the content of the signal much more than on the compression algorithm itself (relatively, because the absolute difference between both algorithms can be seen). To make a general statement, testing more compression algorithms based on various principles is necessary. It may be one of the challenging parts of compression general knowledge.

### Testing of the compression algorithms on short (10 s) signals (CSE database)

In case of SCyF algorithm, the domain (single cycle of ECG) is stored as a part of compressed data and thus, it occupies relatively more memory capacity for short signals. It is also shown in Table 2 that it is quite big difference between quality of signals from CSE database compressed using SPIHT and SCyF algorithms; the difference is probably caused by the short length of signals. For other databases, the difference is not that big.

### Comparison of the results on the whole database and the database without two signals with pacemaker peaks (CSE database)

In Table 2, there are results for the whole CSE database (SCyF P and SPIHT P) and for comparison the results for the CSE database without signals 67 and 70 which include pacemaker peaks. In case of WT + SPIHT algorithm, the results are very similar for both datasets. On the other hand, in case of SCyF algorithm, the results are better when the signals 67 and 70 are excluded. The SCyF algorithm is not tuned for pacemaker signals with high frequencies, because in this variant it uses downsampling of the domain. The results are concordant with this. This example also shows that selecting of only some signals from the database may lead to distortion of the results and may hide the shortcomings of the algorithm.

### Comparison of the results with other authors (MITADB)

As the MITADB is the most used ECG database, results of our new SCyF algorithm tested on this database seem to be comparable with other published algorithms. Results of various compression algorithms based on various principles are included in Supplementary Table S1 online. The Supplementary Table S1 online is divided into 3 parts—3 groups of compression algorithms. In the first group, there are algorithms tested properly on the whole MITADB (48 signals, 2 leads) with subtracted DC component. The second group includes algorithms properly tested (DC subtraction and/or use of PRDN) on the part of the MITADB. In the third group, there are algorithms which (a) testing conditions (such as concrete signals, leads, subtraction of DC) are not properly described in the articles; (b) are tested on any other database excluding MITADB; c) are not correctly tested. The third group is clearly the major one.

There is a lack of standardization in the area of testing and evaluating the performance of compression algorithms. To compare various algorithms developed by various authors, it is necessary to use standard databases as well as standard process of evaluation efficiency and quality of the compression. Alternatively, it is necessary to use the same data without any difference in baseline as the compared author used^54^. Nowadays, the evaluation of efficiency as well as evaluation of the quality of the signal after compression and reconstruction diverge. Also, the selection of the databases or even some signals from one database is non-objective. The most cited database is MITADB; it is also most commonly used for testing in the area of compression. However, many authors select only some signals, one lead or shorten the signals. Tiwari^42^ pointed out this problem and we have to agree. This way of testing is not suitable for any comparison. It is possible that authors select the signals according to the results they reached or fine-tuned their algorithms only on the selected signals. This means that not all of the pathologies and ECG human to human variability are considered. Rakshit^55^ wrote that in most studies, the upper channel (usually lead II) of MITADB is used. He also said that the upper channel is the more quality one. In some studies, the type of database or the details about testing signals are not available at all. The Supplementary Table S1 online shows that most compression algorithms are tested on a part of MITADB. The second problem is inconsistency in subtracting the DC component and offset which can cause different results of quality of the signal after compression and reconstruction. The third problem is inconsistency in methods used for ECG signal quality assessment. Many authors use PRD method which is not correct in case of offset and/or DC presence. This problem is discussed in detail in our previous study^15^. There may be the same problem with other quality assessment methods because some of them are less or more sensitive to DC and offset presence/subtraction (as discussed in^15^).

Thus, the proper comparison of our algorithm with previously published algorithms except for WT + SPIHT tested by us is not possible. It can be done at a rough guess only. In the first group of methods in the Supplementary Table S1 online, there are only our newly published algorithm and algorithm based on WT and SPIHT tested by us. These two algorithms were compared within this study. Only these two algorithms were properly tested. Further, it seems that SCyF method is comparable with methods from the second group at a rough guess. In the third group, only methods highlighted in red are theoretically comparable with SCyF method. In practical way, it is quite difficult, because these methods (excluding DIF + WT + THRQ + RLE) reach high compression ratio balanced with high PRDN. Our method was not tested in this range, because we believe that the distortion will be highly visible even by eye and thus, the compression is pointless. The best results are reached using DIF + WT + THRQ + RLE method, where the CR is 44 and PRDN = 5.87% (the authors declare that all 48 signals were used, but their length and lead are not specified).

It is wort mentioning that there exist MIT-BIH Compression Test database^47,56^ which includes various signals (clean, noisy, physiological, pathological) dedicated for compression, but the database is used only sporadically^57^. In case of lossless methods only once according to Tiwari^42^.

The development of (not only) compression algorithms will be faster if standard databases are used properly and codes are publicly available for future development and comparison. Similar idea was recently published by Tiwari^42^ as well.

One of the limitations of our study is the fact, that not all compression methods ever published are considered. Nevertheless, from the Supplementary Table S1 online it is evident the trend that none of the authors test their algorithms properly; it means testing the algorithm on the whole standard database (without any shortening or selecting of signals or leads), using proper quality assessment algorithms and/or subtracting the offset and/or DC and describing all the details about testing in the published articles.

### Comparison of the original fractal-based algorithm with the proposed SCyF algorithm (MITADB)

Results for original fractal-based algorithm by Ibaida^25^ implemented by us with as little changes as possible are in Table 2. This algorithm was tested on MITADB using two versions of reconstruction. In the first one (Fract 10), for reconstruction of the signal, 10 iterations were used. In case of the second one (Fract 4), only 4 iterations were used as recommended in the original article. The original algorithm fails when compressing signals no. 234 from the first lead and 102 and 220 from the second lead. In the resulting signal, impulses of 10^5^ × higher magnitude than the signal appear. For example, for the second lead of signal 220, PRDN = 390.377% for 10-iteration reconstruction. Thus, these signals are not included in the mean results for Fract 10 and Fract 4 in Table 2 because such a huge error will distort normal results. Using all the signals, the testing would be objective in terms of using the whole database but will distort the mean results of the majority of the signals. Anyway, the original method implemented by us provides much worse results (in terms of both efficiency and quality after compression) than new SCyF method and WT + SPIHT method.

Current version of SCyF algorithm needs manual demarcation of representative single cycle of ECG, which is one of the limitations of the algorithm. This fact has no influence on the results of this method for the purpose of this article and the block of automatized demarcation of single ECG cycle can be added using any QRS detector or delineation algorithm.

### Testing of the algorithms on high-frequency high-resolution signals (UPT signal)

The results of SCyF and SPIHT algorithms tested on high-frequency (5000 Hz) high-resolution (24 bps) signals are shown in Table 2. These signals enable the most efficient compression from tested signals for both compression algorithms; avL = 0.4460 bps and CF ≐ 54 for SCyF algorithm without smoothing. Also, the distortion is the lowest from the Table 2. It means, there exists high redundancy in signals with high sampling frequency and resolution. On the other hand, in this study the influence of compression on high-frequency components of the ECG was not taken into account, which may be considered as the limitation of the study. To compare SCyF with SPIHT, their results are very similar; according to most indexes, SPIHT is slightly better, but according to WEDD SWT and PSim SDNN, better results are reached using SCyF algorithm.

### Comparison of compression of 19 leads of high-frequency signal (UPT signal)

19 leads of UPT signal show differences in terms of compression efficiency and quality of the signal after compression and reconstruction. Some leads are compressed with high efficiency (low avL) and low distortion and some vice versa. Supplementary Fig. S2 online (fs = 5000 Hz) and Supplementary Fig. S3 online (fs = 125 Hz) show the same trend in compression of each lead using two different algorithms. This proves our thought stated above—compression performance is probably dependent on the content of the signal much more than on the compression algorithm itself.

### Testing of how sampling frequency influences compression performance (UPT signal)

**Figure 4:**
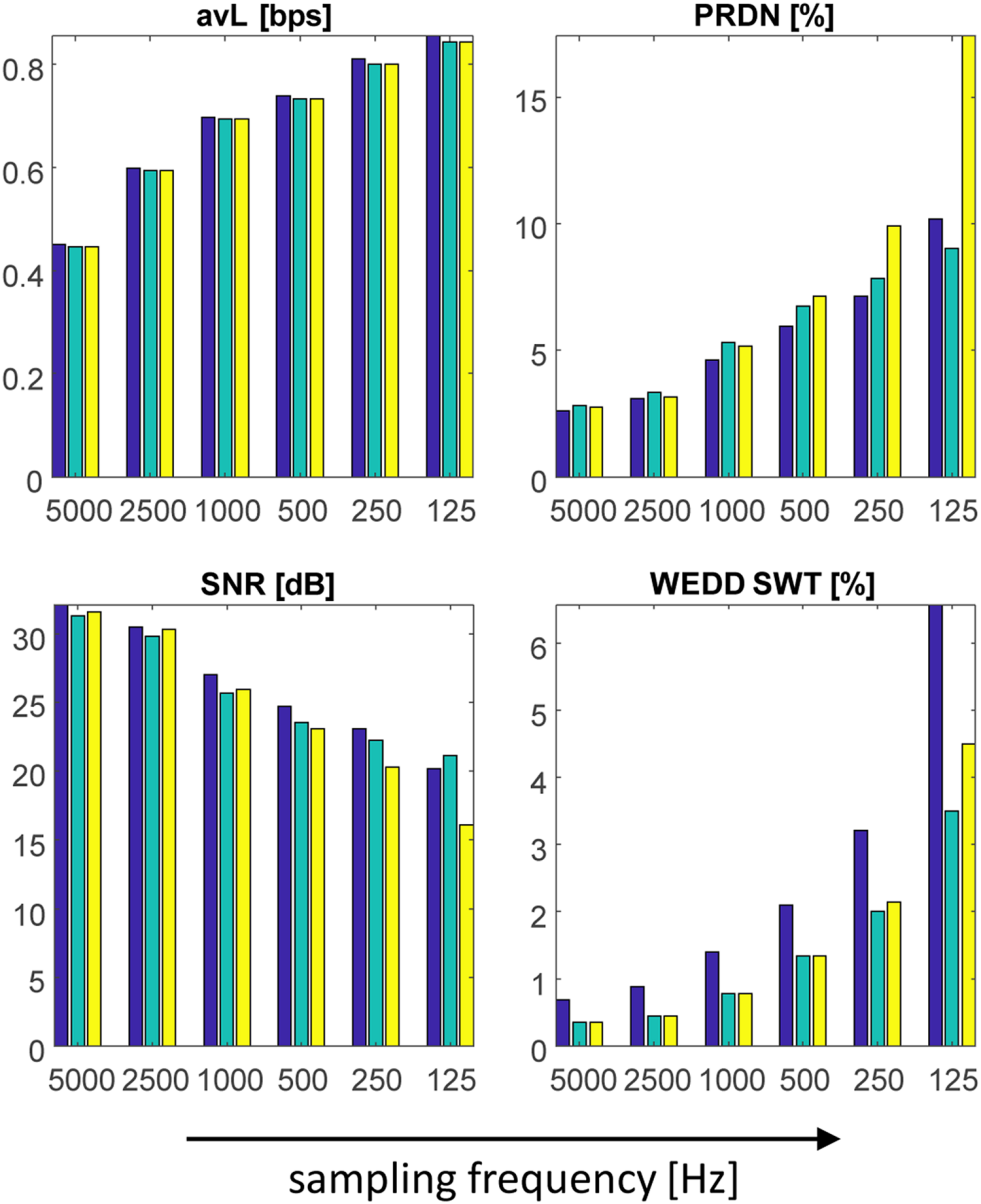
The results of testing WT + SPIHT and SCyF algorithms on UPT signal using 6 various values of sampling frequency. Blue color represents the results of WT + SPIHT algorithm, green and yellow colors represent results of SCyF method without and with smoothing, respectively. For clarity, only 4 representative graphs were selected; complete results are in Supplementary Fig. S4 online.

The higher the sampling frequency, the higher the efficiency of compression (lower avL), lower distortion and higher similarity with original signal using the same settings (except for the length of the domain, which was adjusted to be always one cycle of ECG). The only exception in assessment methods is MAX, where the values changed independently of sampling frequency. Both algorithms have similar trend of compressing signals (it is valid for all sampling frequencies) as can be seen in Supplementary Fig. S2 and Supplementary Fig. S3 online. But the same leads report different results and trends for different sampling frequencies. In case of higher frequencies, the SPIHT algorithm performs better, and in case of lower frequencies SCyF algorithm reaches better results as illustrated in Fig. 4, Supplementary Fig. S4 online and Table 2. According to WEDD SWT, the SCyF algorithm performs better for all sampling frequencies. These findings lead to the summary: higher sampling frequency enables more efficient and more precise compression of ECG signals.

### Testing of the algorithms on free-living data (BUT QDB)

**Figure 5:**
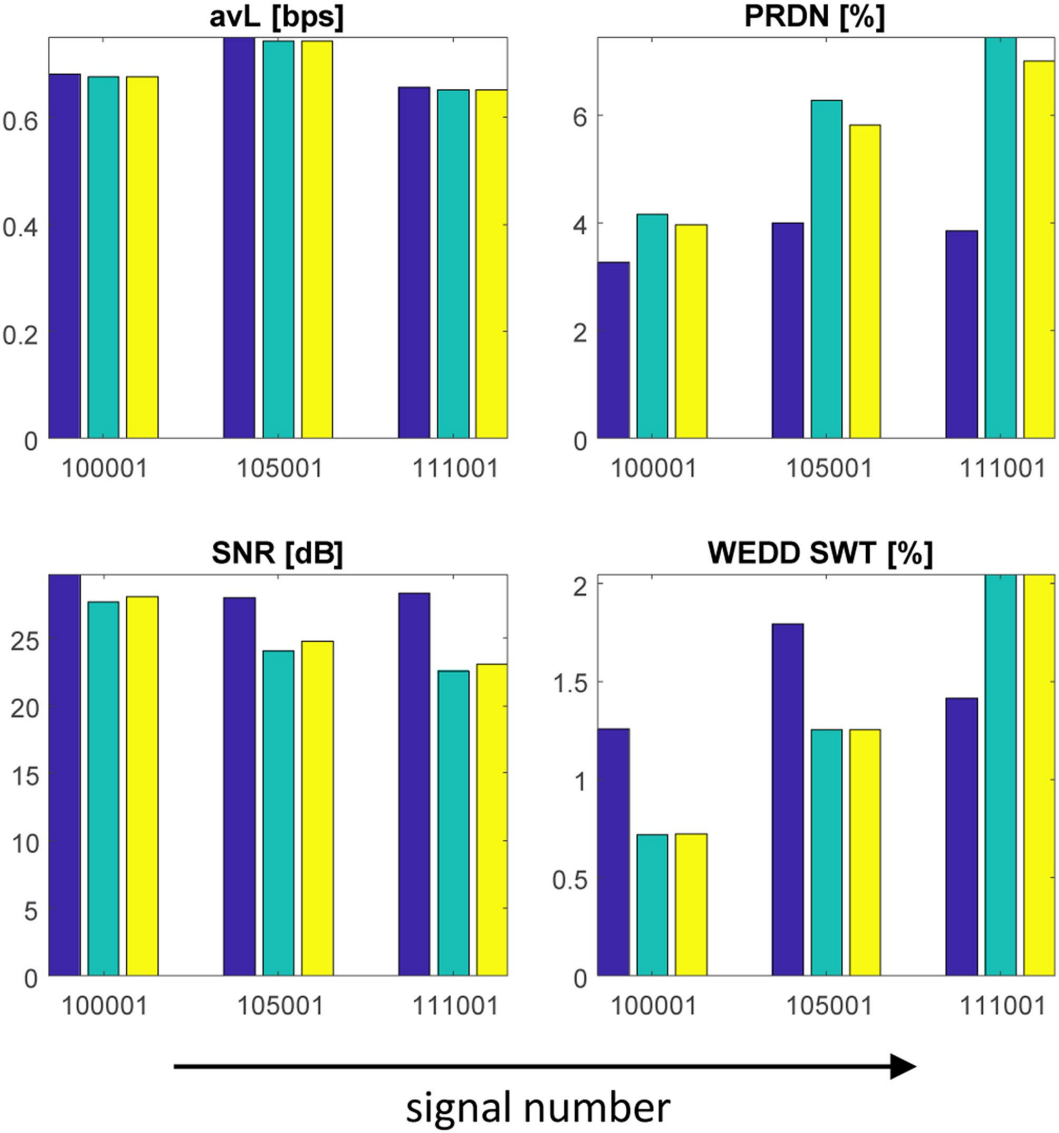
The results of SPIHT and SCyF compression algorithms tested on the BUT QDB. Blue color represents the results of WT + SPIHT algorithm, green and yellow colors represent results of SCyF method without and with smoothing, respectively. For clarity, only 4 representative graphs were selected; complete results are in Supplementary Fig. S5 online.

The algorithms tested on free-living data sampled at 1000 Hz reached good results although free-living signals contain the highest amount of noise from all the databases tested within this study. The avL is in the range 0.6–0.8 bps and quality/distortion metrics reach in most cases better results than in case of standard databases. As can be seen in Fig. 5 and Supplementary Fig. S5 online, SPIHT algorithm outperforms SCyF in most cases except for WEDD SWT in signals 100001 and 105001, PSim SDNN in signal 105001 and SiP10 in signal 100001 where the SCyF algorithm shows better results. In case of signal 111001 which is of the lowest quality from these three signals, the delineation algorithm^58^ was not able to delineate it, thus SiP10 and SiPA10 metrics are not calculated.

### Comparison of compression performance in terms of the original signal quality (BUT QDB)

According to the signal annotations, the percentage representation of each group was calculated and is shown in Table 3. Signal no. 100001 is the most quality one, 105001 is a little bit less quality but on the other hand it contains only 0.03% of quality group (QG) 3. Signal no. 111001 is of the lowest quality and includes 4.43% of QG 3.

**Table 3:** Percentage representation of quality groups in signals from BUT QDB. QG stands for quality group.

Signal	QG 1 (%)	QG 2 (%)	QG 3 (%)
100001	68.64	31.11	0.25
105001	63.45	36.52	0.03
111001	41.94	53.62	4.43

Using the same settings of SCyF algorithm for all three signals, the results are different as can be seen in Fig. 5 and Supplementary Fig. S5 online. The avL is slightly higher for signal no. 105001. In terms of quality, the most quality signal after compression is the signal no. 100001 which is the best quality signal according to Table 3. According to most metrics, the signal no. 111001 is the worst one which also corresponds to the initial quality of the signal. According to some quality assessment methods, compression algorithms tested on signal no. 105001 perform worse quality than in 111001. It can be caused by inter-individual differences in ECG signals. These results lead to the statement that the efficiency and quality of the compression is probably dependent on the initial quality of the signal. This statement should be supported with more testing in the future to be a general knowledge.

### Summary of the findings

This study introduces new compression method—SCyF which is based on fractals and uses single-cycle of ECG as a domain. Compared to the method on which the SCyF algorithm is based, (a) the SCyF uses correct equations for FRMS and fractal scale coefficient calculation; (b) the DC component of the signal is always subtracted before compression; (c) in SCyF algorithm single cycle of ECG is demarcated and stored as a domain to enable reconstruction in one iteration; (d) it enables RB division to enable reaching higher quality of the signal after compression and reconstruction; (e) SCyF algorithm includes optional smoothing block which can improve the quality of the signal in some cases. On MITADB, SCyF algorithm beats the original fractal-based method in terms of efficiency as well as quality.

SCyF algorithm and for comparison also popular and advanced WT + SPIHT based compression algorithm were properly tested on four different databases: two standard databases—CSE and MITADB and two experimental databases—BUT QDB and UPT signal. Twelve different methods for assessment of signal quality after compression, namely PRDN, MSE, SNR, STDERR, MAX, WEDD SWT, PSim SDNN, QS, Spectrum, SiP10, SiPA10, and DTW pmfp2 were used. SCyF algorithm in some cases (especially on UPT signals) and according to some assessment methods (especially WEDD SWT) beats or is comparable with SPIHT algorithm. Nevertheless, the SPIHT algorithm remains the better one in most cases.

## Conclusion

New knowledge and challenges in the area of ECG compression were introduced within this study. Compression performance is probably dependent on the content of the signal much more than on the compression algorithm itself. The absolute results of both algorithms differ but the algorithms have the same trend of performance for each lead. SCyF algorithm performs better on longer-duration signals because it needs to store one-cycle domain. High-frequency high-resolution UPT signals enable the most efficient compression from tested signals for both compression algorithms; avL = 0.4460 bps, CF ≐ 54 and low-level distortion can be reached using SCyF algorithm without smoothing. The sampling frequency influences the performance of compression; the higher the sampling frequency, the better results in terms of both efficiency and quality. SCyF and SPIHT algorithms tested on free-living BUT QDB reach good results (avL of 0.6–0.8 bps and better quality than in standard databases in most cases) although free-living signals contain the highest amount of noise from all the databases tested within this study. Efficiency and quality of the compression are probably dependent on the initial quality of the signal.

None of the authors mentioned in this study tested their compression algorithm properly. Thus, this study also highlights several general and essential problems in the area of compression. It is very difficult to compare reached efficiency and quality of compression with other authors because of the inconsistency in testing conditions. To enable objective comparison of compression algorithms, it is necessary: a) use standard databases of ECG signals and use all the signals in that database without selection of signals or leads and without shortening; b) subtract the DC component before compression; c) make the use of quality assessment methods consistent and meaningful; d) do not use only PRD or its normalized variant for quality assessment; e) describe testing process in detail in articles. Testing of our newly published SCyF algorithm as well as previously published algorithm based on WT and SPIHT serve as an example how the standardization should look like.

## Supplementary information

Supplementary Information.

## Data Availability

The dataset MITADB analyzed during the current study is available in the PhysioNet repository, https://physionet.org/content/mitdb/1.0.0/. The dataset BUT QDB analyzed during the current study is available in the PhysioNet repository, https://physionet.org/content/butqdb/1.0.0/. CSE database analyzed during the current study are available from prof. Paul Rubel, INSERM ERM107—MTIC Hôpital Cardiologique, 28 avenue du Doyen Lépine, 69677 BRON Cedex, France but restrictions apply to the availability of these data, which were used under license for the current study, and so are not publicly available. Data are however available from the authors upon reasonable request and with permission of prof. Paul Rubel, INSERM ERM107—MTIC Hôpital Cardiologique, 28 avenue du Doyen Lépine, 69677 BRON Cedex, France. UPT signal analyzed during the current study is available from the Institute of Scientific Instruments of the Czech Academy of Sciences and from International Clinical Research Center, St. Anne’s University Hospital but restrictions apply to the availability of these data, which were used under license for the current study, and so are not publicly available. Data are however available from the authors upon reasonable request and with permission of the Institute of Scientific Instruments of the Czech Academy of Sciences and International Clinical Research Center, St. Anne’s University Hospital.
