# Supplementary material for: Complex study on compression of ECG signals using novel single-cycle fractal-based algorithm and SPIHT
Source: Sci Rep. 2020 Sep 25;10:15801. doi: 10.1038/s41598-020-72656-6 (PMC7519154; doi:10.1038/s41598-020-72656-6)
Supplement: Supplementary file 1 — Supplementary Information. [file 41598_2020_72656_MOESM1_ESM.pdf]

Complex study on compression of ECG signals using novel single-cycle fractal-based algorithm and SPIHT

Andrea Nemcova[1]*, Martin Vitek[1], Marie Novakova[2]

[1]Department of Biomedical Engineering, Faculty of Electrical Engineering and Communication, Brno University of Technology, Technická 12, 616 00 Brno, Czech Republic

[2]Department of Physiology, Faculty of Medicine, Masaryk University, Kamenice 753/5, 625 00 Brno, Czech Republic

*Corresponding author:

Andrea Nemcova

nemcovaa@feec.vutbr.cz

Supplementary Table S1

*Table S1: Review of compression algorithms based on various principles divided into 3 groups according to the correctness of their testing. *PRDN was calculated as PRDN3 in my review; **baseline and mean were removed for compression but the mean was added for performance evaluation.*

Complex study on compression of ECG signals using novel single-cycle fractal-based algorithm and SPIHT																
Andrea Nemcova, Martin Vitek, Marie Novakova																
**Method**	**First author**	**Ref.**	**Year**	**Database**	**Signals**	**offset/DC**	**CF**	**PRD**	**PRDN**	**SNR**	**RMS**	**QS**	**CC**	**WEDD**	**WWPRD**	**WDD**
Algorithms correctly tested on the whole MITADB																
SCyF	Nemcova	-	2019	MITADB	48, both leads	no/yes	10,41<br>avL = 1,06		7.15	24.05		4.16		4.02		
WT+SPIHT (tested by us)	Hrubes	[17]	2008	MITADB	48, both leads	no/yes	10.31<br>avL = 1.07		6.36	25.39		4.86		3.67		
Algorithms correctly tested on the part of the MITADB																
WT+SPIHT	Hrubes	[17]	2008	MITADB	100-121, lead I (each 10 min)	?	8<br>avL = 1,37		5.26							
WT+THRQ+RLE	Agulhari	[18]	2013	MITADB	100, 101, 102, 103, 107, 109, 111, 115, 117, 118, 119, 213, 222, 232 (each 10 min)	no/yes	9.21	4.06								
SCAE	Wang	[39]	2019	MITADB	48, lead I, (200 beats)	no/yes	106.45	8.00		22.79	0.03	16.44				
Algorithms not correctly tested, with lack of information or tested on differnt DB																
PCA+CS+HC	Singh	[30]	2016	PTB	12 standard leads	?	13.91	5.03		23.99				1.82	6.8	
				CSE	12 standard leads	?	10.39	9.55		21.4				5.88	20.72	
CS+k-LiMapS	Adamo	[29]	2015	MITADB	100, 101, 102, 109, 111, 112, 113, 115, 117, 119, 121	no	39.97	0.79	14.14	39.71						
LPF+downsampling	Elgendi	[9]	2018	MITADB	48, lead ?	?	6	1.88								
EMD + encoding	Khaldi	[32]	2012	?	1	?	59	6.1								
EMD+skeleton	Zhao	[33]	2016	MITADB	100, 101, 115, 118, 121, 123, 220, lead I	no/yes	18.34		6,91*							
AFD+symbol subst.	Ma	[36]	2015	MITADB	48, lead I	no	25.64	1.05	16.14	16.3	0.22					
JPEG2000	Bilgin	[19]	2003	MITADB	104, 107, 111, 112, 115, 116, 117, 118, 119, 201, 207, 208, 209, 212, 213, 214, 228, 231, 232 (each 1 min)	yes/no	20	5.17								
					100, 101, 102, 103, 107, 109, 111, 115, 117, 118, 119 (each 10 min)	yes/no	20	3.26								
DIF+WT+THRQ+RLE	Jha	[21]	2016	MITADB	48, lead ?	no	44	0.36	5.87	59.5	3.53	143				
WT+SPIHT	Lu	[13]	2000	MITADB	100, 101, 102, 103, 107, 109, 111, 115, 117, 118, 119 (each 10 min)	yes/no	20	6.49								
					104, 107, 111, 112, 115, 116, 117, 118, 119, 201, 207, 208, 209, 212, 213, 214, 228, 231, 232 (each 1 min)	yes/no	20	7.52								
skeleton	Fira	[37]	2008	MITADB	100, 101, 102, 103, 104, 105, 106, 115, 117, 118, 119, 201, 202, 205, 207, 208, 209, 212, 213, 214, 215, 217, 219, 232 (each 10,000 samples)	no	18.27	1.17	17.37		11.35					
CT+HC	Lee	[14]	2011	MITADB	all (one lead)	no	21.3	1.75	24.93	13.1	16.74	15.89				
Fractal-based	Khalaj	[24]	2009	MITADB	100, 102, 103, 104, 105, 108, 109, 117	?	14.66	9.8					98.33			
Improved fractal-based	Lin	[11]	2015	MITADB	100, 101, 102, 103, 104, 105, 106, 107, 108, 109, 111, 112, 113, 114, 117, 118, 119 (each 10 s)	?	42	1.7								
Fractal-based (cloud)	Ibaida	[25]	2014	MITADB	?	?	42	< 1								
ML WT+SVD+THR	Padhy	[22]	2016	PTB	12/15 leads	no/yes	22.1	6.93						2.8		2.67
EMD+WT	Wang	[34]	2016	MITADB	100, 105, 108, 109, 112, 121, 202, 207, lead ? (each 2 min)	yes/yes	23.02	6,11**								
Blaschke unwinding AFD	Tan	[38]	2019	MITADB	48, lead I	no/no	35.53	1.47		31.05		32.58				
WT+THRQ+QUANT	Rebollo-Neira	[54]	2019	MITADB	48, lead ?	no/no	23.17	0.53	8.08			43.93				
EMD+WT	Jha	[35]	2020	MITADB	48, lead ? (1 minute)	no/no	21.56	4.65	6.80	54.17		5.38 (PRD)<br>3.27 (PRDN)				
DCT + JPEG2000	Pandey	[20]	2020	MITADB	48, lead I (2 minutes)	no/no	19.48	1.01	16.76	16.3	9.73	19.88 (PRD)				

Supplementary Figure S1

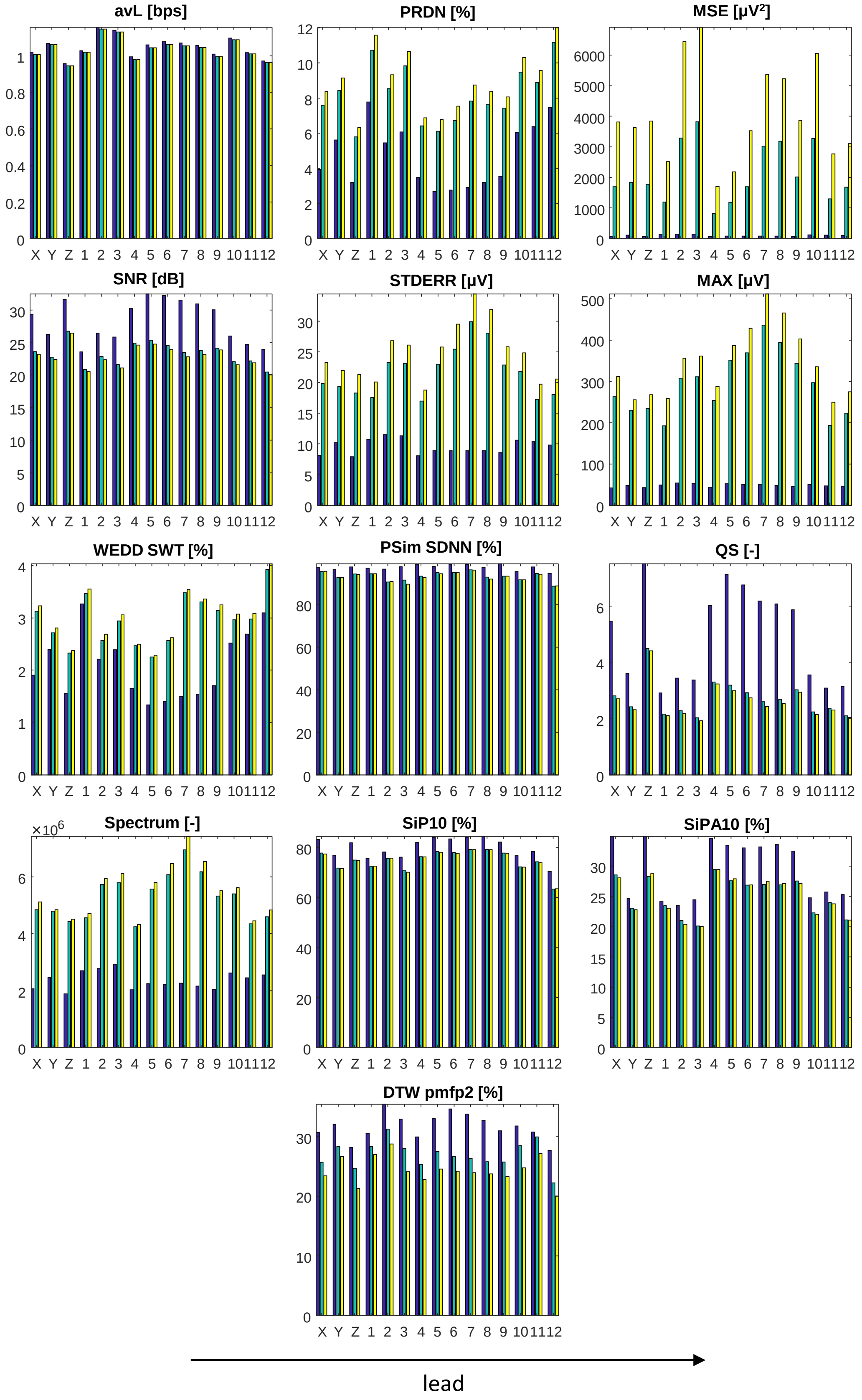

Supplementary Figure S1: Complete results of testing WT+SPIHT and SCyF algorithms on the 15 leads of signals from the CSE database including signals 67 and 70. Blue color represents the results of WT+SPIHT algorithm, green and yellow colors represent results of SCyF method without and with smoothing, respectively.

Supplementary Figure S2

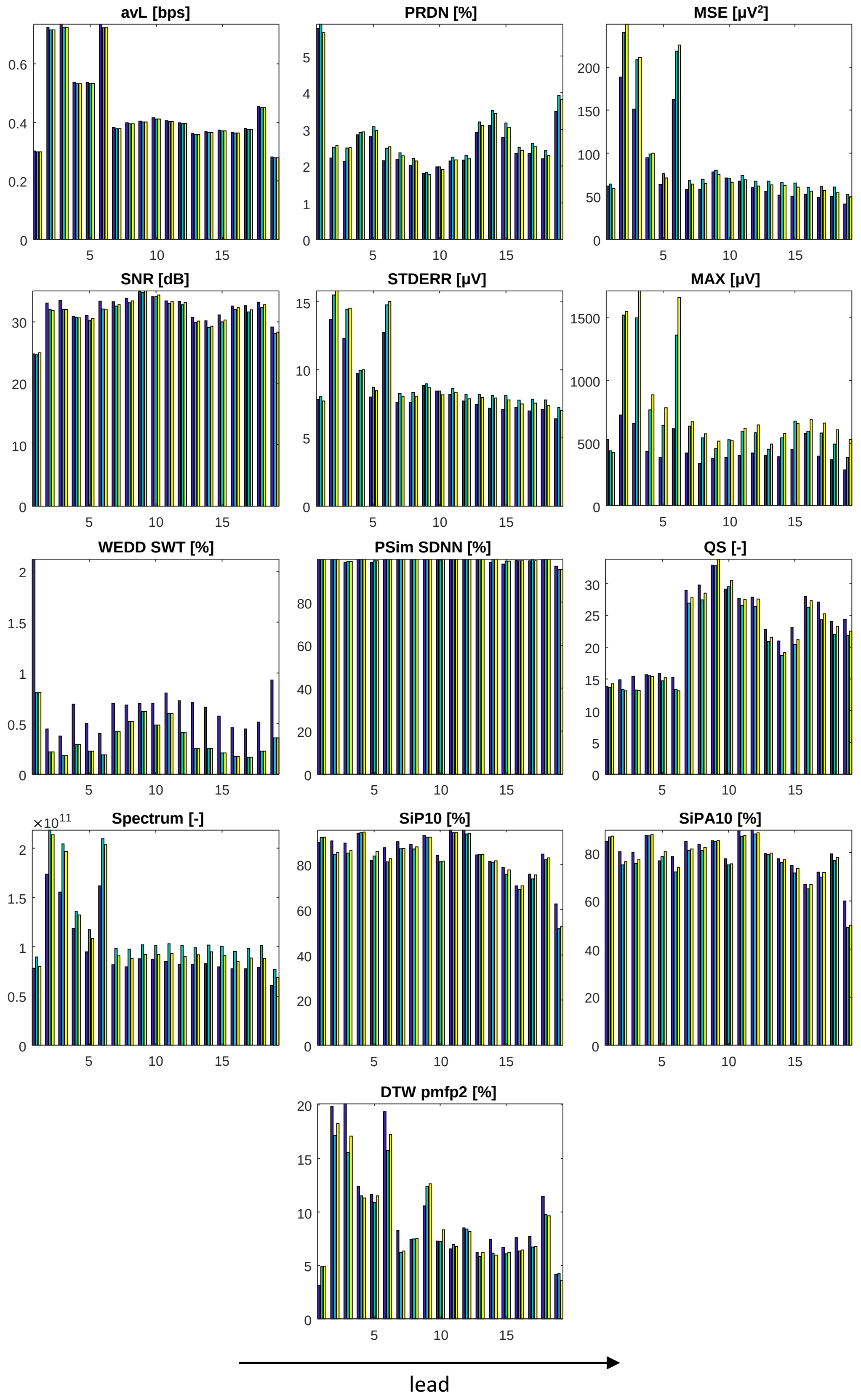

Supplementary Figure S2: Complete results of testing compression algorithms based on WT + SPIHT and SCyF using 19 leads of UPT signal. The sampling frequency of the signals is 5,000 Hz. Blue color represents the results of WT+SPIHT algorithm, green and yellow colors represent results of SCyF method without and with smoothing, respectively.

Supplementary Figure S3

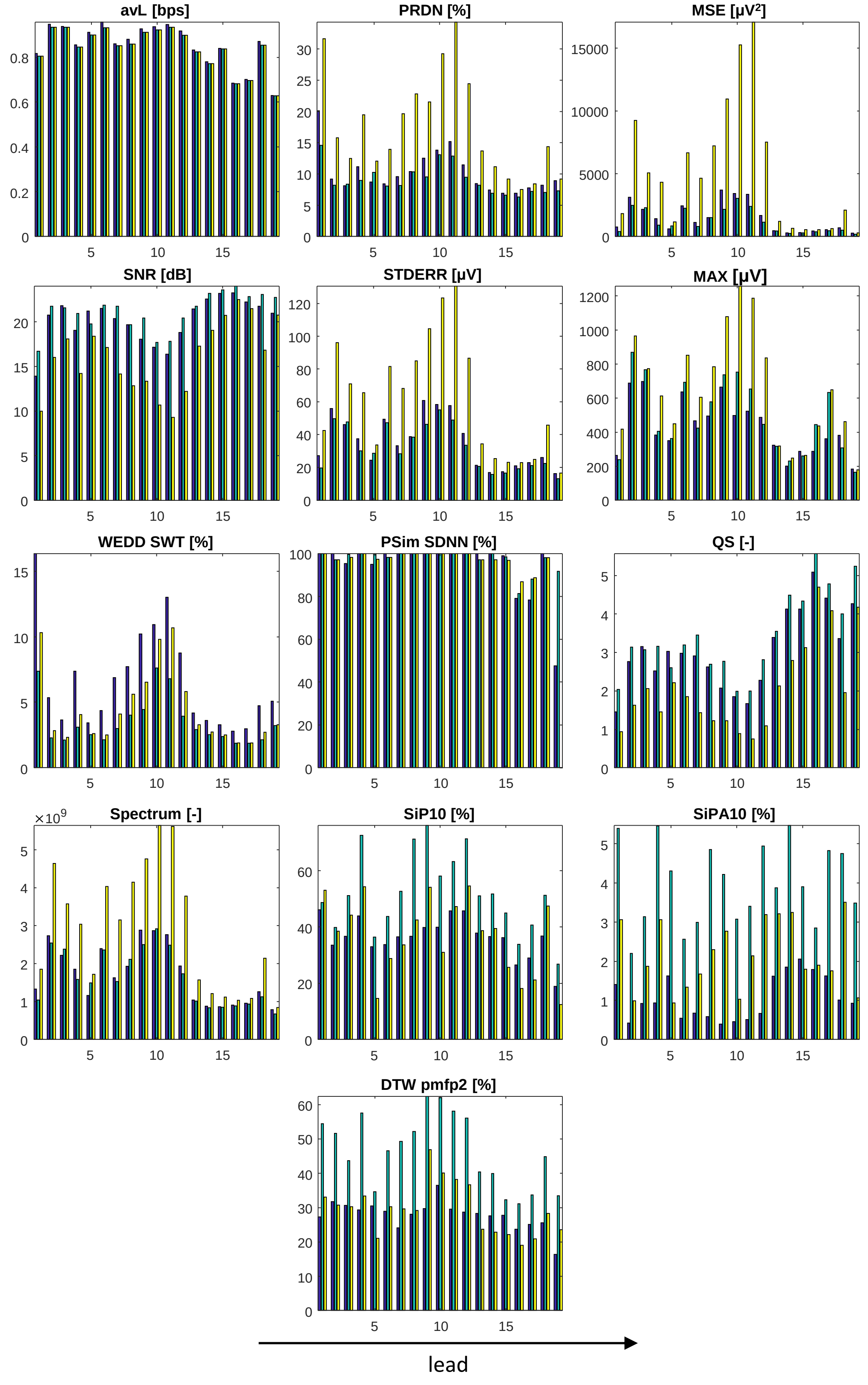

Supplementary Figure S3: Complete results of testing compression algorithms based on WT + SPIHT and SCyF using 19 leads of UPT signal. The sampling frequency of the signals is 125 Hz. Blue color represents the results of WT+SPIHT algorithm, green and yellow colors represent results of SCyF method without and with smoothing, respectively.

Supplementary Figure S4

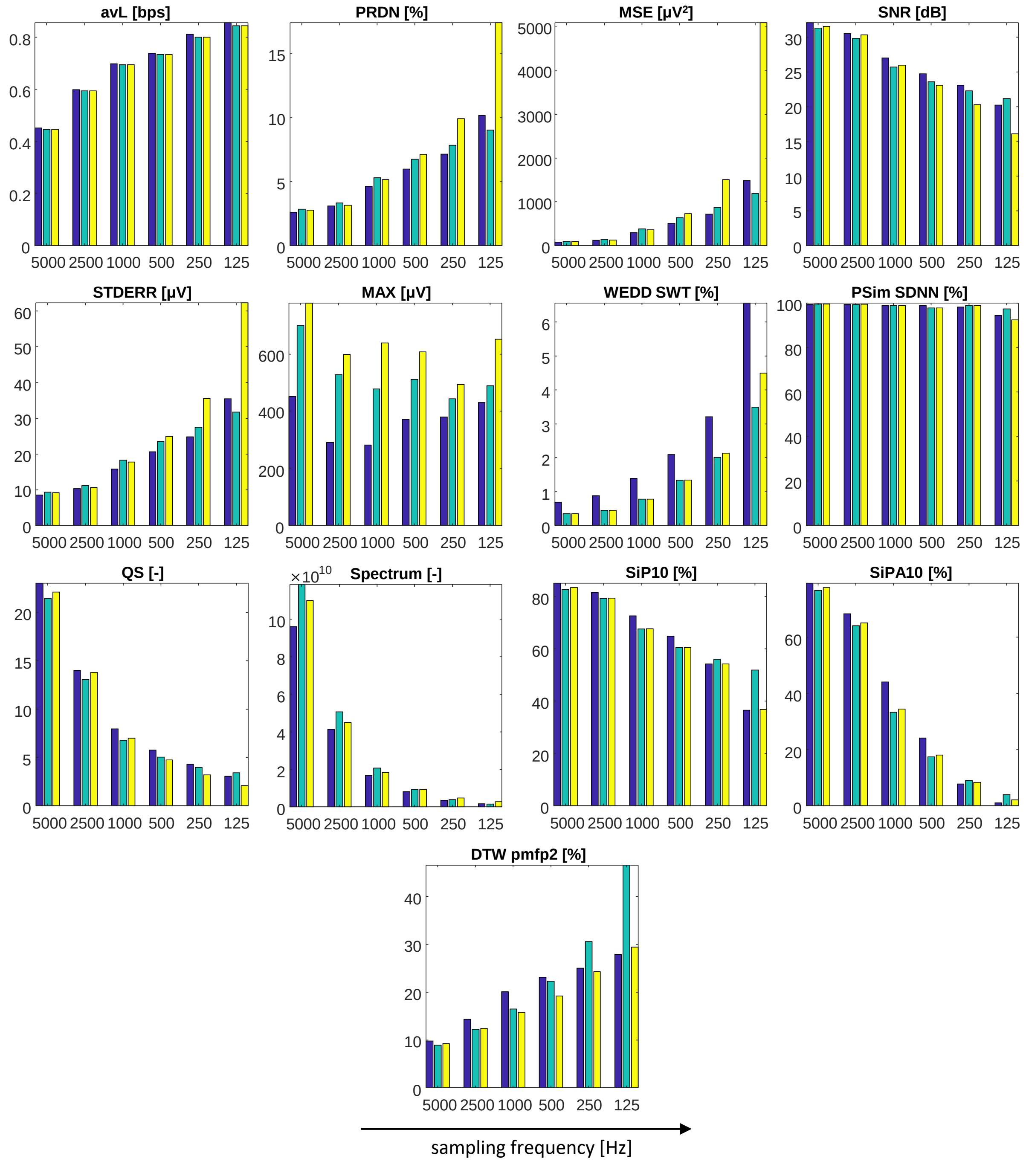

Supplementary Figure S4: Complete results of testing WT + SPIHT and SCyF algorithms on UPT signal using 6 various values of sampling frequency. Blue color represents the results of WT+SPIHT algorithm, green and yellow colors represent results of SCyF method without and with smoothing, respectively.

Supplementary Figure S5

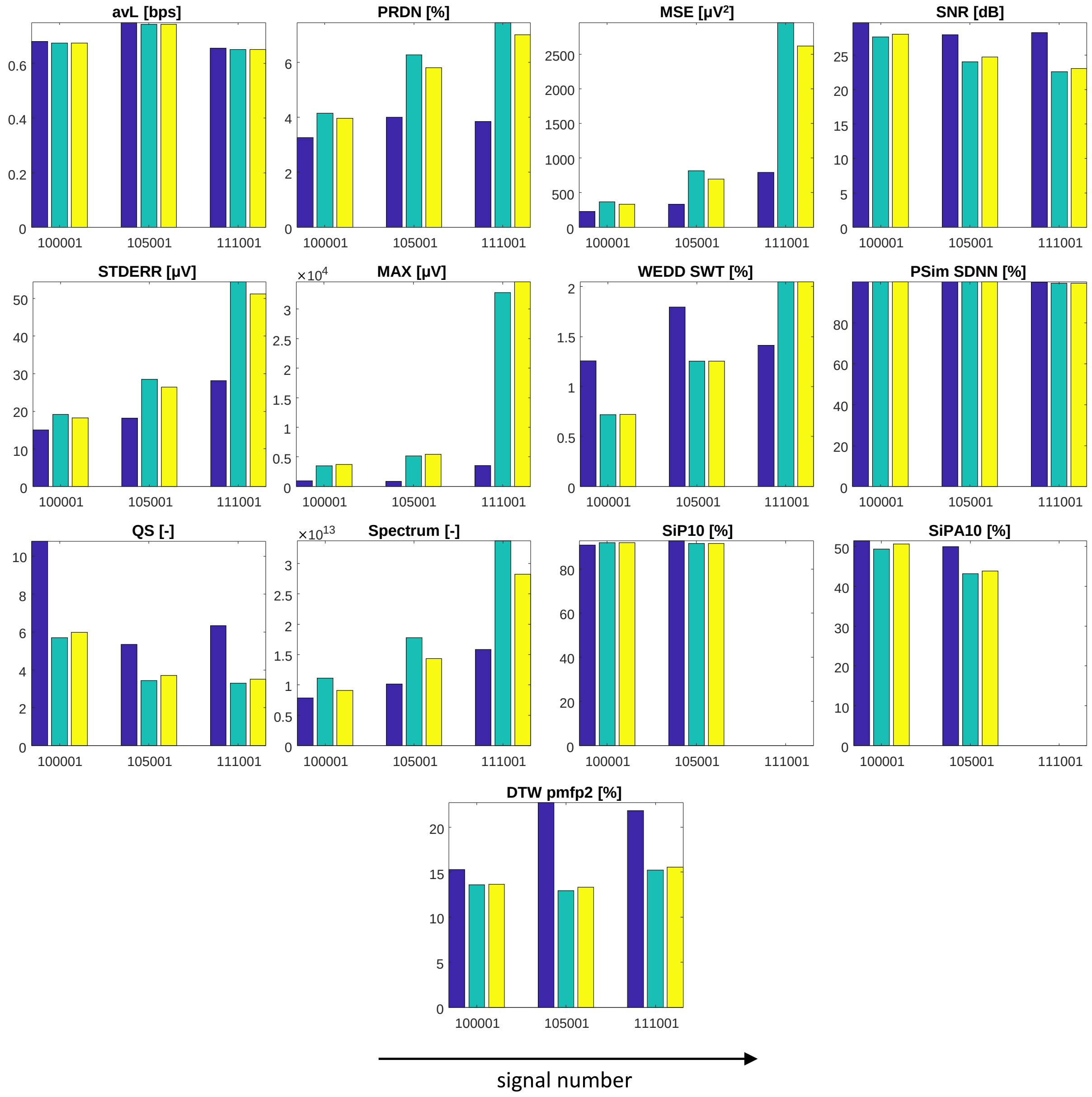

Supplementary Figure S5: Complete results of SPIHT and SCyF compression algorithms tested on the BUT QDB. Blue color represents the results of WT+SPIHT algorithm, green and yellow colors represent results of SCyF method without and with smoothing, respectively.
